# Microbiomes and chemical components of feed water and membrane-attached biofilm in reverse osmosis system to treat membrane bioreactor effluents

**DOI:** 10.1038/s41598-018-35156-2

**Published:** 2018-11-14

**Authors:** Tomohiro Inaba, Tomoyuki Hori, Hidenobu Aizawa, Yuya Sato, Atsushi Ogata, Hiroshi Habe

**Affiliations:** Environmental Management Research Institute, National Institute of Advanced Industrial Science and Technology (AIST), 16-1 Onogawa, Tsukuba, Ibaraki, 305-8569 Japan

## Abstract

Reverse osmosis (RO) system at a stage after membrane bioreactor (MBR) is used for the wastewater treatment and reclamation. One of the most serious problems in this system is membrane fouling caused by biofilm formation. Here, microbiomes and chemical components of the feed water and membrane-attached biofilm of RO system to treat MBR effluents were investigated by non-destructive confocal reflection microscopy, excitation-emission fluorescence spectroscopy and high-throughput sequencing of 16S rRNA genes. The microscopic visualization indicated that the biofilm contained large amounts of microbial cells (0.5 ± 0.3~3.9 ± 2.3 µm^3^/µm^2^) and the extracellular polysaccharides (3.3 ± 1.7~9.4 ± 5.1 µm^3^/µm^2^) and proteins (1.0 ± 0.2~1.3 ± 0.1 µm^3^/µm^2^). The spectroscopic analysis identified the humic and/or fulvic acid-like substances and protein-like substances as the main membrane foulants. High-throughput sequencing showed that *Pseudomonas* spp. and other heterotrophic bacteria dominated the feed water microbiomes. Meanwhile, the biofilm microbiomes were composed of diverse bacteria, among which operational taxonomic units related to the autotrophic *Hydrogenophaga pseudoflava* and *Blastochloris viridis* were abundant, accounting for up to 22.9 ± 4.1% and 3.1 ± 0.4% of the total, respectively. These results demonstrated that the minor autotrophic bacteria in the feed water played pivotal roles in the formation of polysaccharide- and protein-rich biofilm on RO membrane, thereby causing membrane fouling of RO system.

## Introduction

Wastewater reclamation is one of the most common and serious issues in the world. A dual-membrane treatment system combining the microfiltration (MF) or ultrafiltration (UF) membrane and the reverse osmosis (RO) membrane is a wastewater reclamation technology that attracts attention due to its high treatment efficiency and high economic benefit^1,2^. In membrane bioreactor (MBR), wastewater is first treated by activated sludge and second passed through MF or UF membranes^3^. The MBR effluent is further treated by RO system to generate reclaimed water. Actually, the MBR-RO system has been applied to municipal wastewaters^4^.

Despite the effectiveness of the MBR-RO system, fouling of RO membrane reduces the productivity and increases the energy costs^5^. A causative factor of membrane fouling is the biocake build-up or biofilm formation. A laboratory experiment using model bacteria showed that biofilm formation on RO membrane caused the decrease in the RO permeate flux^6^. On the other hand, in actual water treatment plants, membrane fouling is known as a complex phenomenon that occurs in association with activities of microbiomes^7–9^. Yet there is little knowledge about the microbiological mechanisms underlying membrane fouling at naturally occurring states. So far, it has been only known that some of microbial species were frequently found on the fouled RO membrane^10^. The specific localization and proliferation of microorganisms on RO membrane should be one criterion for their involvement in membrane fouling. Thus, direct comparison of microbiomes of the feed water and membrane-attached biofilm in RO system allows the identification of the membrane fouling-causing microorganisms.

The objective in this study was to investigate the microbiomes and chemical components of the feed water and membrane-attached biofilm in a pilot-scale RO system. Before the RO membrane treatment, the synthesized wastewater (SWW) and heavy oil-containing SWW were treated by MBR and the resultant effluents served as the feed water of RO system. A combined approach of microscopy, spectroscopy and high-throughput sequencing was firstly implemented to better understand the microbiological and chemical mechanisms of RO membrane fouling. The three-dimensional structure and component of the membrane-attached biofilm were clarified by non-destructive confocal reflection microscopy. The types of chemical components were identified by excitation-emission fluorescence spectroscopy. Furthermore, the feed water microbiome and the membrane-attached biofilm microbiome revealed by 16S rRNA gene sequencing were directly compared to identify the microorganisms associated with membrane fouling of RO system.

## Methods

### Preparation of MBR effluents as the feed water of RO system

A pilot-scale MBR was operated to prepare the effluents. The schematic image of the MBR and the detailed experimental settings were reported previously^11,12^. Briefly, the pilot scale (230-L) MBRs were started up with an inoculum of activated sludge obtained from a municipal wastewater treatment plant (Kinu aqua-station, Ibaraki, Japan). The flow rates of both the SWW input and effluent output were 115 L/day, resulting in the hydraulic retention time (HRT) of 2 days. The excess sludge was not produced and MLSS showed ranges of 5,100–8,000 and 6,450–12,300 mg/L during the treatments of SWW and heavy oil-containing SWW, respectively. An SWW with the following composition was fed continuously to the reactor: CH_3_COONa (2.65 g/L), NH_4_Cl (0.376 g/L), KH_2_PO_4_ (0.109 g/L), peptone (0.706 g/L), FeCl_3_ 6H_2_O (0.782 mg/L), CaCl_2_ (1.56 mg/L), MgSO_4_ (1.56 mg/L), KCl (1.56 mg/L) and NaCl (1.56 mg/L). As for the heavy oil-containing SWW treatment, a commercially available heavy oil (Japanese Industrial Standards (JIS) number K2205) was added at a flowing rate of 2.3 g/day using a syringe pump (Pump 11 Elite, Harvard Apparatus, Holliston, US). The amounts of the residual organic compounds in the MBR effluents of SWW and heavy oil-containing SWW were measured. Briefly, the total organic carbon (TOC) concentrations in the MBR effluents were analyzed using a TOC analyzer (TOC-L; Shimadzu, Kyoto, Japan). The average TOC concentrations of the MBR effluents of SWW and heavy oil-containing SWW during the RO system operation showed 11.3 and 20.7 mg/L, respectively, indicating the organic carbon was effectively removed during the MBR treatment. Nevertheless, it cannot be excluded that some of microorganisms passed through the MF membrane (pore size: 0.07 μm) installed in the MBR. Each MBR effluent was stored in a tank at room temperature, which suggested the potential that the passing and indigenous microorganisms grew during the storage. The MBR effluents were used as the feed water of RO system.

### Operation of a pilot-scale RO system treating MBR effluents

A pilot-scale RO system (Membrane master RUW-5CH; Nitto Denko Matex, Aichi, Japan) with a membrane module (Nitto denko, Osaka, Japan) equipping spiral membrane element (LFC3-LD-D2, Nitto denko) was operated. The membrane element was made by composite polyamide, and the membrane active area was 37.1 m^2^. The schematic view of RO system is shown in Supplementary Fig. S1. The system was assembled with unused and/or clean parts, although the whole system was not sterilized. There is a possibility that some of microorganisms inhabited the component parts of system. Each operation was started with an outflow rate of 0.165 L/min. In order to accelerate the RO membrane fouling, the brine water was returned to the feed water storage tank. The RO membrane treatment continued until the amount of outflow fell below the set value and the decreased outflow rate did not recover by the increase in pump pressure. The ammonium ion (NH_4_^+^) and nitrate ion (NO_3_^−^) concentrations, in addition to the TOC concentration, of the feed and RO treated waters were monitored throughout the operation by a capillary electrophoresis system (CE; Agilent, Santa Clara, CA, USA). Furthermore, the concentrations of sulfate ion (SO_4_^2−^), phosphate ion (HPO_4_^2−^) and chloride ion (Cl^−^) of the feed water were determined at the start and end points of the operation by the capillary electrophoresis system, while those of sodium, magnesium and calcium were determined at the same time points by an inductive coupled plasma spectrometer (SPS 3000S, Seiko Instruments, Tokyo, Japan). The concentrations of salts in the feed water are shown in Supplementary Table S1. The salinity levels of the feed water were similar as those of brackish water (0.05–3%). At the end of the operation, the fouled RO membrane element was dismounted from the membrane module and stored at 4 °C before the microscopic observation, fluorescence spectroscopy and high-throughput sequencing.

### Biofilm visualization and quantitative imaging analysis

The fouled RO membrane samples were observed by the direct confocal reflection microscopy (CRM)^13–15^. Briefly, a confocal laser scanning microscope (LSM880; Carl Zeiss, Jena, Germany) equipped with a 40 × /0.80 numerical aperture water immersion objective (Achroplan W; Carl Zeiss) was used to acquire the biofilm images. For live/dead staining, the RO membrane samples were treated for 15 minutes with SYTO9 for live cells and Propidium iodide (PI) for dead cells (Molecular Probes, OR, US) at final concentrations of 5 µM and 15 µM, respectively. The other staining dyes ConA-FITC conjugate (Sigma-Aldrich, MO, USA), FM4-64 (Molecular Probes) and FilmTracer SYPRO Ruby Biofilm Matrix Stain (Molecular Probes) were used to visualize polysaccharides, lipids (plasma membrane) and proteins, respectively, according to the manufacturers’ instructions. The fluorescent dyes-treated RO membrane samples were washed with water once and set in the sample chamber filled with water in order to wash out the non-specific binding dye and residual dye. For non-destructive observation, the fouled membrane samples were visualized using water immersion lens without a coverslip. Reflected light of 514-nm argon laser was used for reflection microscopy. Illumination with 453-, 488- and 514-nm argon lasers was conducted to detect the SYPRO Ruby, SYTO9/ConA-FITC and FM4-64 fluorescence, respectively. The PI fluorescence was detected with a 543 nm He-Ne laser. An MBS T80/R20 filter (Carl Zeiss) was used as the main beam splitter to detect the reflected light. The obtained images were analyzed using the ZEN software (Carl Zeiss) for the three-dimensional image construction. At least six microscopic images at random points on the RO membrane were taken per sample and the representative images are shown. The total amount, biofilm thickness, cell amount, living cell rate, the polysaccharide, lipid and protein amounts were determined based on six to ten independent observations, and the standard deviations are indicated. The amounts of polysaccharides, lipids and proteins were determined based on the ConA-FITC, FM4-64 and SYPRO Ruby fluorescent signals. The cell amount and living cell rate were calculated from the fluorescent signal intensities of SYTO9 and PI. The image analysis of biofilm was performed using Comstat2 program (www.comstat.dk)^16,17^.

### Three-dimensional excitation-emission fluorescence spectroscopy

Three milliliter of feed water and 200-mm^2^ piece of fouled RO membranes were analyzed by three-dimensional excitation-emission fluorescence spectroscopy. The measurement of each sample were performed three times and the representative spectrum is shown. The RO membranes were immersed in 0.1 M citric acid or NaOH solution (pH 12.0) for 24 hours to extract foulants, and then the citric-acid extract and neutralized NaOH extract were centrifuged to separate the solid and liquid fractions. The supernatants of the acid and alkali extracts were subjected to the spectroscopy. Three-dimensional excitation-emission matrixes (3D-EEMs) of the feed water and foulants were obtained using a spectrofluorometer (AquaLog; Horiba, Kyoto, Japan) with a 10 mm quartz cell. The details of 3D-EEM measurement using AquaLog was described in a technical report of Horiba Jobin Yvon^18^. Briefly, the experimental set up of 3D-EEM measurement is as follows: integration time 0.5 sec, 220–800 nm excitation spectra at 3 nm increments, 245.21–827.61 nm emission spectra, 8-pixel (4.6 nm) emission increment and medium CCD gain. The Raman scatter influences were eliminated by subtracting the EEM of ultrapure water (18.2 Ωcm^−1^) from that of the samples. To reduce scatter from the water Raman peak, all EEMs were corrected through the blank (ultrapure water) subtraction according to the emission and excitation correction factors provided by the manufacturer. The signal intensities in regions where first and second order Rayleigh scattering appeared were also nullified by the AquaLog software (Horiba). The primary and secondary inner filter effects were corrected using the UV-vis absorbance spectra.

### DNA extraction, PCR amplification and high-throughput Illumina sequencing

In order to collect the planktonic microorganisms being present in the feed water of RO system, the feed water was filtered through nitrocellulose membranes with pore sizes of 0.22 and 0.05 µm (MF-millipore, Merck). In each collection, 0.7 to 1.0 L of the feed water was filtered, and the membrane was subjected to DNA extraction. For the fouled RO membrane, approximately 100-mm^2^ pieces of the membrane samples were taken and stored at −20 °C until DNA extraction. The sample pieces were cut from inner region of spiral membrane. Total DNA was extracted from the feed water and membrane-attached biofilm samples according to a direct-lysis protocol that included bead-beating^19^, and detailed procedures were described in previous report^13^. Three replicates were conducted for the DNA extraction and the subsequent procedures for the feed water and biofilm samples. The PCR amplification with the universal primers targeting the V4 region of 16S rRNA genes^20,21^ was performed as described previously^13^. High-throughput Illumina sequencing was performed according to the previously mentioned procedure^22^. Briefly, the PCR products were purified with an AMPure XP kit (Beckman Coulter, CA, USA) and then with a QIAquick gel extraction kit (Qiagen, Venlo, Netherlands). The concentrations of purified PCR products were quantified with a Quant-iT PicoGreen dsDNA reagent (Life Technologies, CA, USA) and a NanoDrop 3300 (Thermo Fisher Scientific). An appropriate amount of the purified fragment and an internal control (PhiX Control V3; Illumina, CA, USA) was subjected to paired-end sequencing with a 300-cycle MiSeq reagent kit (Illumina) and a MiSeq sequencer (Illumina). The removal of the PhiX control, chimeric and low-quality (Q < 30) sequences and the assembling of the paired-end reads were performed following the procedure reported previously^23^. The sorted sequences in each library were phylogenetically characterized using the QIIME software^24^. OTU was defined using the cut-off of 97% sequence identity. The closest relative of the OTU was determined by NCBI BLAST search (http://blast.ncbi.nlm.nih.gov/). Alpha-diversity indices (i.e., Chao1, Shannon and Simpson reciprocal) and the weighted UniFrac distances for the principal coordinate analysis (PCoA) were calculated based on an equal number (n = 25,894) of sequences using the QIIME software^24^. The α-diversity index Chao1 indicates the predicted total number of microbial species (i.e., richness), whereas the Shannon and Simpson reciprocal indices reflect the evenness and richness of microbiomes with a focus on the minor and dominant microorganisms, respectively^25^. The raw sequence data obtained in this study were deposited in the sequence read archive in the DDBJ database (http://www.ddbj.nig.ac.jp/) under the accession number DRA006513.

## Results and Discussion

### Physicochemical profile during the treatment of MBR effluents by RO system

Physicochemical profiles of the feed water and RO membrane-treated water are shown in Supplementary Figs S2 and S3. At the beginning of the operation of RO system, the TOC concentrations of the RO membrane-treated water showed 5.5 mg/L for the MBR effluent of SWW and 9.0 mg/L for the MBR effluent of heavy oil-containing SWW. In order to facilitate the membrane fouling, the brine water of the RO treatment was recirculated to the feed water storage tank. Due to this reason, the concentrations of organic carbon and nitrogen compounds in the feed water increased with time. The changes in operating pressure during the RO treatment are also shown in Supplementary Figs S2 and S3. At the final phase of each operation, the flow rates of the RO membrane-treated water were not recovered by increasing the inlet pressure. Simultaneously with the decrease in the treated water volume, the diminished quality of the treated water was observed, indicating that the serious fouling occurred in RO system.

### Three-dimensional structure of the biofilm on the fouled RO membrane

**Figure 1:**
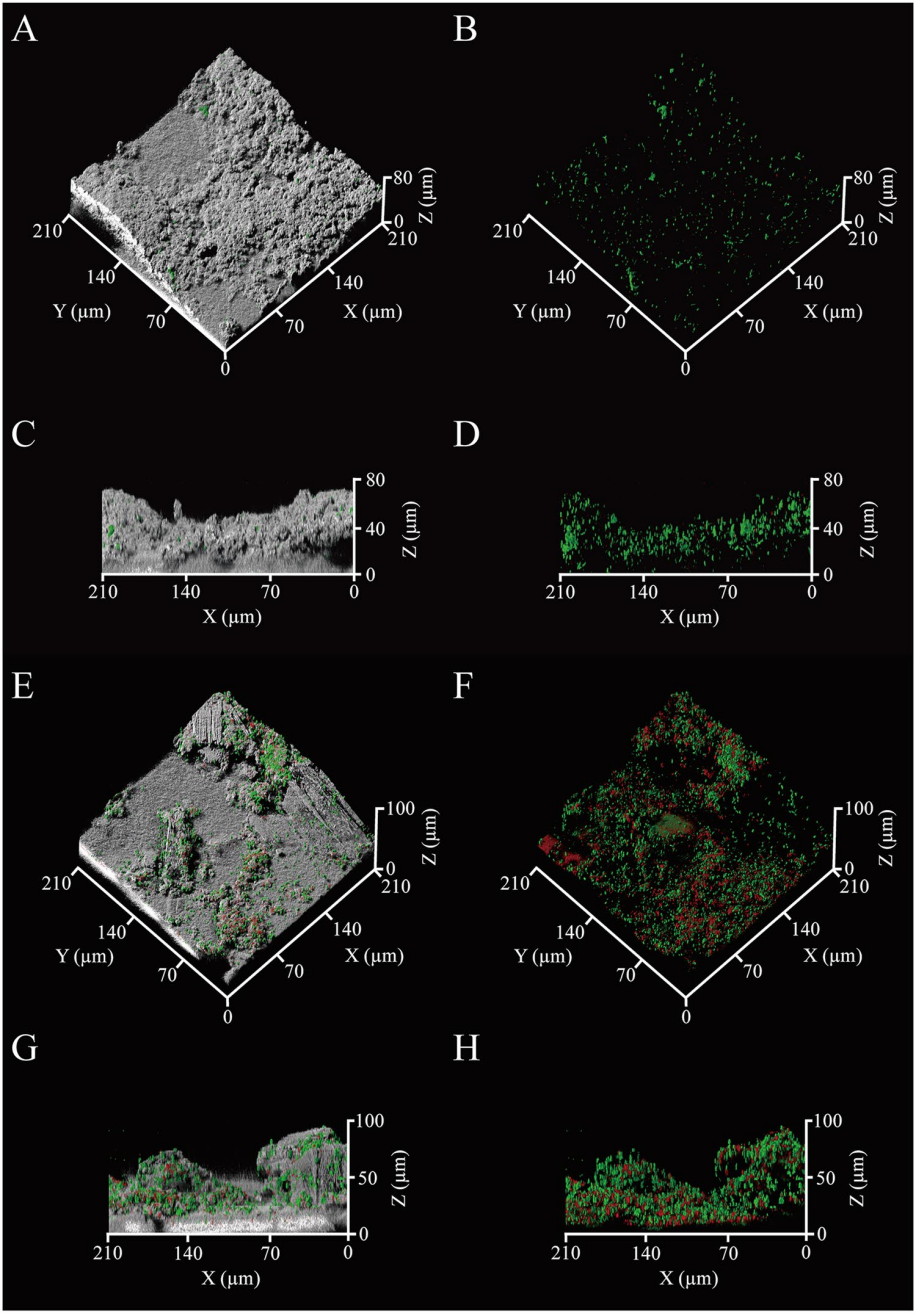
The three-dimensional structures of membrane-attached biofilms. The gray areas indicate the physical body reflected by light, while green and red indicate living and dead microbial cells, respectively. The panels show the biofilms formed in RO system to treat the MBR effluents of the SWW (**A**–**D**) and heavy oil-containing SWW (**E**–**H**). The fluorescent images of same region are also shown (**B**,**D**,**F**,**H**). At least 6 microscopic images at random points were taken per a sample and the representative images are shown.

**Figure 2:**
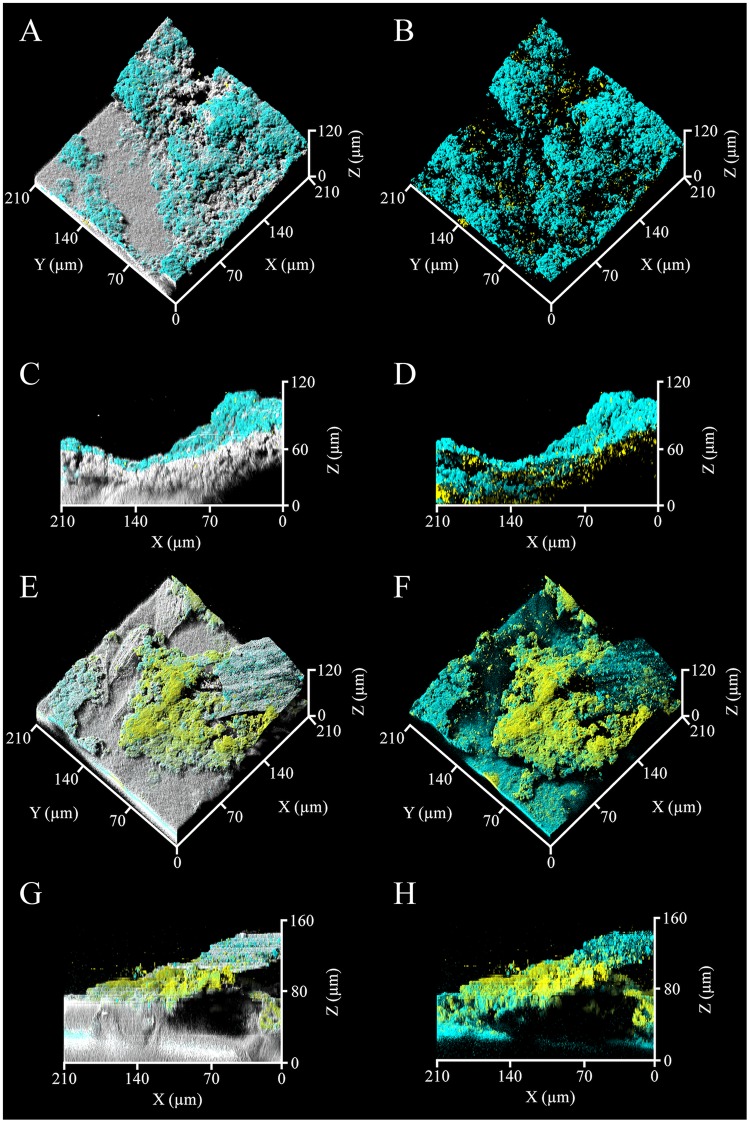
Extracellular matrixes of the membrane-attached biofilms. Cyan and Yellow indicate polysaccharides and lipids probed by ConA-FITC and FM4-64, respectively. Gray indicates the physical body reflected by light. The panels show the biofilms formed in RO system to treat the MBR effluent of the SWW (**A**–**D**) and heavy oil-containing SWW (**E**–**H**). The fluorescent images of same region are also shown (**B**,**D**,**F**,**H**). At least 6 microscopic images at random points were taken per a sample and the representative images are shown.

The CRM was applied for non-destructive visualization of the biofilm formed on the fouled RO membrane. When the MBR effluent of SWW was used as the feed water, the SYTO9 and PI staining and quantitative image analysis indicated that most of microbial cells on RO membrane were alive (living cell rate; 81.6 ± 11.7%), but the major part of the biofilm was non-cell region (Fig. 1A–D and Table 1). Subsequently, the ConA-FITC and FM4-64 imaging showed that the non-cell region was mainly composed of polysaccharides, whereas lipids were rather small constituent (Fig. 2B,D). The FM4-64 dye is a probe for the selective detection of lipid bilayer (plasma membrane) of microbial cells. Its signals mostly overlap with signals of the SYTO9 dye (Figs 1B,D and 2B,D) and the amounts of the lipids and microbial cells were comparable (Table 1). Therefore, the lipid signals observed in the biofilm was most likely originated from microbial cells. The SYPRO Ruby dye imaging further indicated that proteins colonized highly in the biofilms (see Supplementary Fig. S4). This dye generally does not stain microbial cells, thus the extracellular proteins also accumulated on the membranes, contributing to the membrane fouling.

**Table 1:** Quantitative image analysis of membrane-attached biofilms.

		SWW^a^	Heavy oil-containing SWW^a^
Total amount	(µm^3^/µm^2^)	78.3 ± 25.2	63.3 ± 5.9
Average thickness	(µm)	88.2 ± 30.2	73.2 ± 8.3
Maximum thickness	(µm)	113.5 ± 39.8	92.0 ± 8.1
Cell amount^b^	(µm^3^/µm^2^)	0.5 ± 0.3	3.9 ± 2.3
Living cell rate^b^	(%)	81.6 ± 11.7	59.4 ± 9.0
Polysaccharide amount^b^	(µm^3^/µm^2^)	9.4 ± 5.1	3.3 ± 1.7
Lipid amount^b^	(µm^3^/µm^2^)	1.1 ± 0.9	2.8 ± 1.4
Protein amount^b^	(µm^3^/µm^2^)	1.0 ± 0.2	1.3 ± 0.1

^a^All data were based on the average of 6 to 10 replicates and the standard deviations are shown.

^b^Significant difference between the SWW and Heavy oil-containing SWW (P < 0.05, *t-*test).

On the other hand, employing the MBR effluent of the heavy oil-containing SWW as the feed water, both of living and dead microbial cells were observed in the biofilm, and their amounts were much larger than those in the biofilm for SWW (Fig. 1E–H and Table 1). In this regard, the lipid signals possibly derived from microbial cells showed higher amounts (Fig. 2E–H and Table 1). The extracellular polysaccharides showed lower amounts compared to that for SWW, but still, they were one of the main components of the biofilm, whereas the proteins were detected at the similarly high levels for SWW (Table 1 and see Supplementary Fig. S4). Consequently, the results of microscopic visualization indicated that the accumulation of living microbial cells and microbially produced substances, especially polysaccharides and proteins, on RO membrane was the primary factor for the decreases in the quality and quantity of the RO membrane-treated water and the incidence of membrane fouling in RO system.

### Three-dimensional EEM fluorescent spectra for the feed water and membrane-attached biofilm

**Figure 3:**
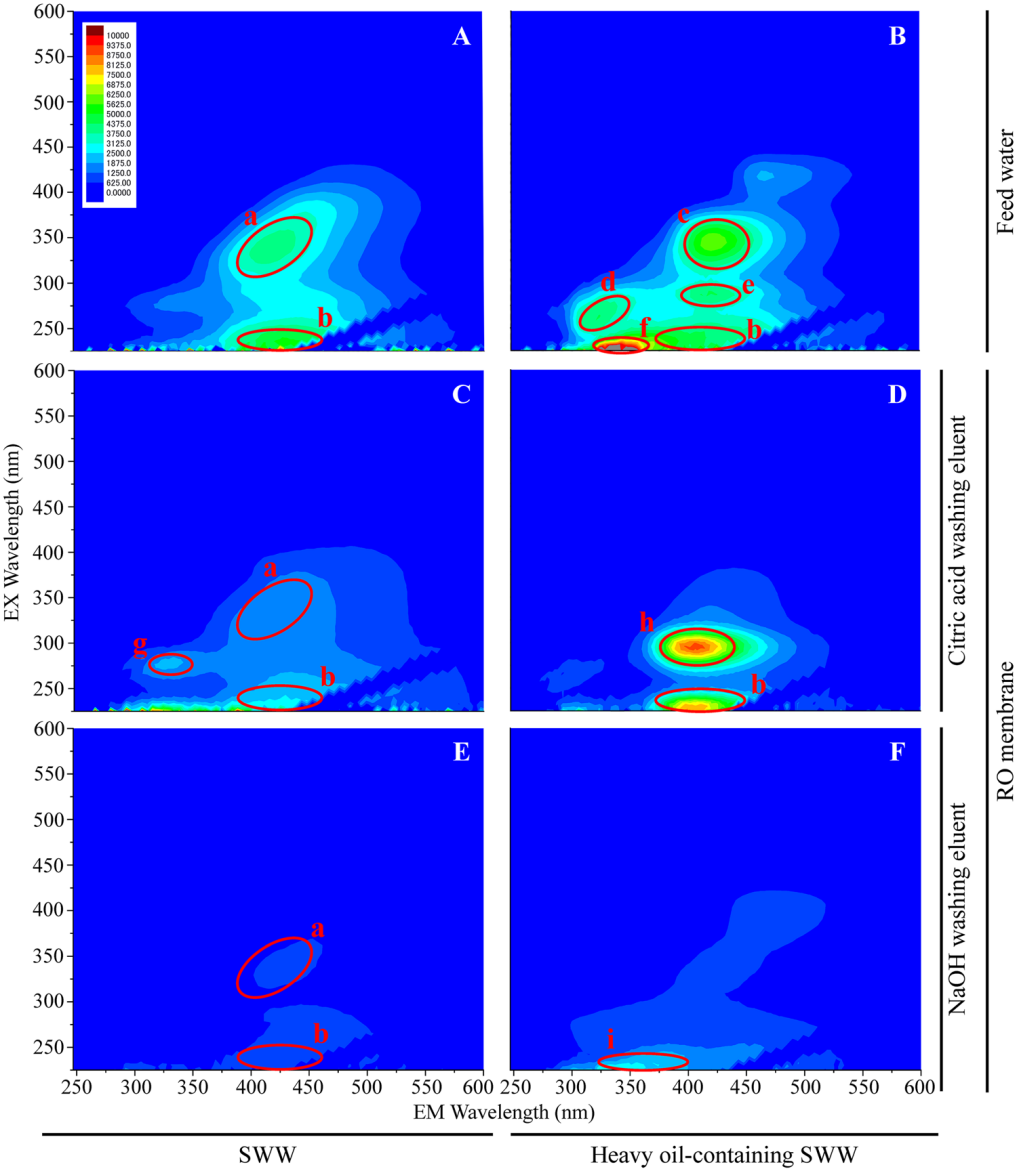
Three-dimensional excitation-emission matrix (3D-EEM) spectra of the feed water and membrane attached-biofilms. The small letters indicate the main peaks. The panels show the spectra of the feed water (**A**,**B**) and membrane-attached biofilms (**C**–**F**). The measurements of each samples were performed 3 times and the representative spectra are shown.

EEM fluorescence spectroscopy was conducted to investigate what kinds of chemical compounds were present in the feed water and membrane-attached biofilm of the system (Fig. 3). In the feed water including MBR effluents, six main peaks were observed. Peak a (Ex/Em of 300–375/375–450 nm) and peak b (Ex/Em of 225–250/370–425 nm) appeared in the MBR effluent of SWW. It has been reported that the peaks a and b were estimated to be humic and fulvic acid-like substances, respectively^26^. In the MBR effluent of heavy oil-containing SWW, there found to be peaks at the Ex/Em wavelengthes of 300–375/380–460 nm (designated peak c), 250–300/300–350 nm (peak d), 275–300/380–450 nm (peak e) and 225–240/330–370 nm (peak f). The peaks c and e were reported to belong to the humic acid-like and marine humic acid-like fractions, respectively^26^. The other peaks d and f were previously assigned to the tryptophan and aromatic protein-like substances^26^.

Meanwhile, it is vital to point out the specific appearance of some peaks only in the membrane-attached biofilms not in the feed water. In the membrane-attached biofilm for the MBR effluent of SWW, peak g was found at the Ex/Em wavelength of 260–280/310–330 nm, which was estimated to be the tryptophan protein-like substance^26^. Peak h (Ex/Em 290–325/380–425 nm) and peak i (Ex/Em 225–250/325–400 nm) were appeared specifically in the biofilm for the MBR effluent of heavy oil-containing SWW. It has been reported that the peak h was defined as marine humic acid-like substance and peak i was defined as aromatic protein-like or hydrophobic acid-like substance^26^. Remarkably, the 3D-EEM spectral analysis showed that the humic and/or fulvic acid-like substances (i.e., the peaks a, b and h) existed abundantly in the membrane-attached biofilms. Humic and fulvic acids are the polymeric substances containing sugar moieties and their accumulation is known to provoke membrane fouling in membrane filtration processes^27,28^. Moreover, the number of detected peaks was larger in the biofilm of heavy oil-containing SWW than that of SWW. It has been previously reported that the presence of oils and their degradative intermediates caused the loss of membrane flux and the severe fouling of membrane filtration processes^29–31^. The spectroscopic result is consistent with the microscopic result, i.e., the primary presence of the ConA-FITC signals that could represent the sugar moieties of humic and fulvic acids, as well as polysaccharides, in the biofilms. In addition, the fluorescence spectroscopy detected protein-like substances (e.g., the peaks g and i) from the biofilms, which is also agreed with the microscopic result (see Supplementary Fig. S4). These results certified the effectiveness of a complementary use of the microscopic and spectroscopic methods to detect broad range of chemical compounds at high sensitivity and with high accuracy. Together, the fluorescence spectroscopy highlighted that the accumulation of humic and fulvic acid-like substances and protein-like substances on RO membrane is essential for membrane fouling of RO system.

### Diversity and dynamics of the feed water and biofilm microbiomes

With the microscopic and spectroscopic results to indicate that membrane fouling of RO system was mainly caused by the accumulation of living microbial cells and microbially produced polysaccharides and proteins, the feed water and membrane-attached biofilm microbiomes were next evaluated by high-throughput Illumina sequencing of 16S rRNA genes. The summary of Illumina sequencing data is shown in Supplementary Table S2. A total of 1,909,526 sequences were obtained in 40 libraries, corresponding to an average of 47,738 sequences per library (minimum: 23,742; maximum: 126,963).

The α-diversity indices of the feed water microbiomes were not notably different between the pore sizes (i.e., 0.22 and 0.05 µm) of cell collection filters, and this trend was common in both the MBR effluents of the SWW and heavy oil-containing SWW. Contrastingly, microbiomes of the membrane-attached biofilms were more diverse than the feed water microbiomes for both the MBR effluents, suggesting the membrane-attached lifestyle, or biofilm, more highly increased the microbiome diversity rather than the planktonic lifestyle.

**Figure 4:**
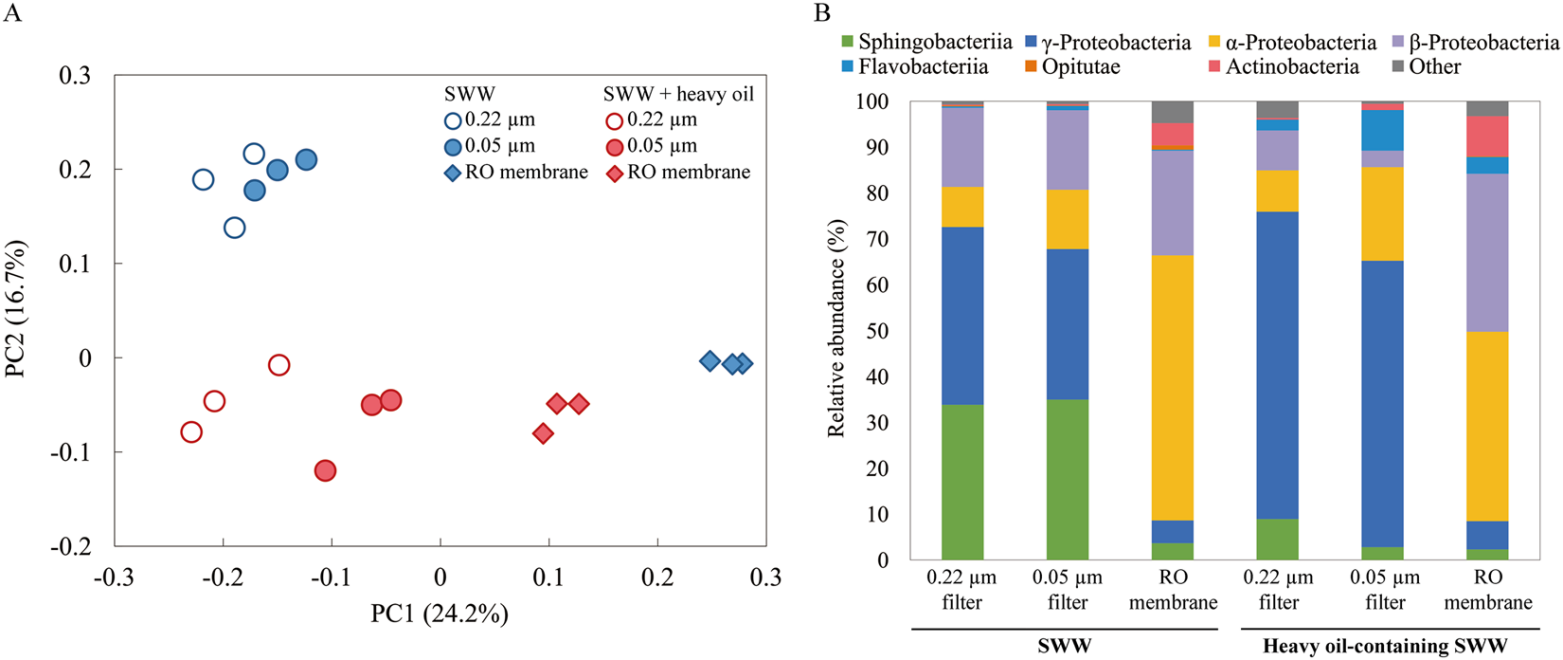
Comparison and compositional changes of the feed water and membrane-attached biofilm microbiomes. (**A**) Principal coordinate analysis (PCoA) scatter plot of 16S rRNA genes obtained from Illumina sequencing. The weighted UniFrac distances were calculated based on an equal number (n = 25,894) of sequences. Open and closed circles indicate the microbiomes of feed water collected on 0.22-µm and 0.05-µm filters, respectively. The closed diamonds indicate the membrane-attached biofilm microbiomes. The blue and red colors of symbols show those for the MBR effluents of the SWW and heavy oil-containing SWW, respectively. (**B**) Class-level distribution of the feed water and membrane-attached biofilm microbiomes. The relative abundances of each bacterial class are shown. All data were based on the average of 3 replicates.

PCoA of the Illumina sequence data based on weighted UniFrac distances was used to compare the whole structures of the microbiomes of the feed water and membrane-attached biofilm (Fig. 4A). For the MBR effluent of SWW, there was little difference in the cell size-separated feed water microbiomes, although the microbiomes collected on the 0.05-µm filter scattered slightly right area from the plots of those on the 0.22-µm filter. For the MBR effluent of heavy oil-containing SWW, the similar trend was found. In addition, the examination at the starting day of the RO treatment indicated that the microbiomes little changed during the periods of the treatment (see Supplementary Table S3 and Fig. S5). Intriguingly, for both the MBR effluents of SWW and heavy oil-containing SWW, the membrane attached-biofilm microbiomes scattered the right area of the PCoA plot and they were located distantly from the feed water microbiomes. In accordance with the data of the α-diversity indices, the PCoA results indicated that microbiomes of the feed water and membrane attached-biofilm were remarkably different from each other.

### Class-level distribution of the feed water and biofilm microbiomes

A class-level phylogenetic analysis of the sequence data was performed to determine the microbiome compositions of the feed water and membrane-attached biofilms (Fig. 4B). For the MBR effluent of SWW, the classes *Sphingobacteriia*, *α-*, *β-* and *γ-Proteobacteira* were the main members of the feed water microbiomes collected with the 0.22- and 0.05-µm filters. For the MBR effluent of heavy oil-containing SWW, the class *γ-Proteobacteria* was the most abundant, accounting for more than half of the total, in the 0.22- and 0.05-µm filter-collected feed water microbiomes. The class *Flavobacteriia* was more abundant than that for SWW. Nevertheless, the feed water microbiomes were similar for both the MBR effluents, and the cell size separation apparently not affected the microbiome compositions. Concerning the membrane-attached biofilm microbiomes, for both the MBR effluents of SWW and heavy oil-containing SWW, the class *α-Proteobacteria* was the most abundant member, accounting for more than 40% of the total. The abundances of the class *γ-Proteobacteria* was much smaller than those of the feed water microbiome. Moreover, especially for the MBR effluent of heavy oil-containing SWW, the class *β-Proteobacteria* showed higher relative abundances in the biofilm microbiome than in the feed water microbiome. The compositions of the feed water and biofilm microbiomes were notably different, and the high dominances of *α-* and *β-Proteobacteria* of the biofilm microbiomes suggested their significant contribution to membrane fouling of RO system.

### Species-level identification of the feed water and biofilm microbiomes

To determine the dominant bacterial species in the feed water and biofilm microbiomes, the 10 most abundant operational taxonomic units (OTUs) were identified (Tables 2 and 3, see Supplementary Tables S4 and S5). The abundant OTUs of the feed water microbiomes for both the MBR effluents of SWW and heavy oil-containing SWW were focused. The OTUs affiliated within the genus *Pseudomonas* were the predominant member, accounted for more than 30% of the total (Tables 2 and 3). It has been reported that *Pseudomonas* spp. were isolated from the surface of RO membrane installed in the MF-RO system treating municipal wastewater^32^ and they were detected from the fouling layer of RO membrane^33^. The OTU 102009 (*Pedobacter composti* [accession no. NR041506, 100% sequence similarity]) was the most abundant in the MBR effluent of SWW, and also it was one of the 5 most abundant OTUs in the MBR effluent of heavy oil-containing SWW. *Pedobacetr* spp. have been detected from water reclamation system and bottled mineral water^34,35^, suggesting that the OTU 102009 could grow under the oligotrophic conditions. Microbial species within the genera *Brevundimons* (OTU 83690) and *Caulobacter* (OTU 119287) were also detected (Table 3 and see Supplementary Tables S4 and S5), and both of them have been detected from RO membrane^36,37^. The OTU-level identification revealed that the heterotrophic bacteria were abundant in the feed water microbiomes.

**Table 2:** The 5 most abundant OTUs in microbiomes of the feed water and membrane-attached biofilm for SWW.

OTU ID		Accession Number	Bacteria		Identity (%)	Relative abundance (%)^a^	Fold changes relative to		
			Class	Species			0.22 µm^a^	0.05 µm^a^	RO^a^
0.22 µm									
	102009	NR041506	*Sphingobacteriia*	*Pedobacter composti*	100	34.2 ± 8.2	—	1.0	16.3
	5095	EU780001	*γ-Proteobacteria*	*Pseudomonas pseudoalcaligenes*	99	24.9 ± 3.3	—	1.7	597.2
	12685	KX980435	*γ-Proteobacteria*	*Pseudomonas anguilliseptica*	100	11.4 ± 2.0	—	1.0	77.2
	164722	KM272838	*β-Proteocbacteria*	*Herbaspirillum huttiense*	93	8.5 ± 0.7	—	0.7	12.1
	74997	KY241478	*β-Proteocbacteria*	*Limnobacter thiooxidans*	100	4.3 ± 0.2	—	1.9	49.5
0.05 µm									
	102009	NR041506	*Sphingobacteriia*	*Pedobacter composti*	100	35.3 ± 3.9	1.0	—	16.8
	5095	EU780001	*γ-Proteobacteria*	*Pseudomonas pseudoalcaligenes*	99	14.6 ± 2.0	0.6	—	350.6
	164722	KM272838	*β-Proteocbacteria*	*Herbaspirillum huttiense*	93	12.0 ± 0.5	1.4	—	17.1
	12685	KX980435	*γ-Proteobacteria*	*Pseudomonas anguilliseptica*	100	11.7 ± 1.1	1.0	—	79.3
	8590	KY249241	*γ-Proteobacteria*	*Pseudomonas veronii*	100	4.0 ± 0.4	18.0	—	208.1
RO membrane									
	163830	AF229876	*β-Proteocbacteria*	*Azoarcus tolulyticus*	100	8.2 ± 1.0	NA	8823.5	—
	98583	NR116005	*α-Proteobacteria*	*Reyranella massiliensis*	100	7.8 ± 0.8	2.4	3.2	—
	35813	KY393035	*β-Proteocbacteria*	*Hydrogenophaga pseudoflava*	100	5.3 ± 0.4	4.3	6.0	—
	171350	NR104697	*α-Proteobacteria*	*Hyphomicrobium vulgare*	99	3.9 ± 0.3	122.0	128.1	—
	137174	KU991464	*α-Proteobacteria*	*Gemmobacter lanyuensis*	100	3.4 ± 0.3	91.3	86.5	—

NA, Not applicable.

^a^All data were based on the average of 3 replicates, and the standard deviations are shown for relative abundances.

**Table 3:** The 5 most abundant OTUs in microbiomes of the feed water and membrane-attached biofilms for the heavy oil-containing SWW.

OTU ID		Accession Number	Bacteria		Identity (%)	Relative abundance (%)^a^	Fold changes relative to		
			Class	Species			0.22 µm^a^	0.05 µm^a^	RO^a^
0.22 µm									
	133530	KY643712	*γ-Proteobacteria*	*Pseudomonas stutzeri*	100	36.6 ± 9.9	—	19.2	1504.4
	42311	KX354320	*γ-Proteobacteria*	*Pseudomonas caeni*	100	21.4 ± 4.0	—	0.4	3680.1
	102009	NR041506	*Sphingobacteriia*	*Pedobacter composti*	100	8.5 ± 0.3	—	41.6	8.1
	83690	KY576007	*α-Proteobacteria*	*Brevundimonas denitrificans*	100	3.0 ± 2.7	—	0.5	0.9
	170668	KM594394	*γ-Proteobacteria*	*Pseudomonas mendocina*	100	2.8 ± 0.1	—	8.2	3.5
0.05 µm									
	42311	KX354320	*γ-Proteobacteria*	*Pseudomonas caeni*	100	56.7 ± 6.8	2.6	—	9735.5
	176899	AB682419	*Flavobacteriia*	*Flavobacterium cucumis*	99	8.5 ± 2.4	13.6	—	47.5
	83690	KY576007	*α-Proteobacteria*	*Brevundimonas denitrificans*	100	6.1 ± 1.0	2.0	—	1.8
	72044	NR125598	*α-Proteobacteria*	*Gemmobacter megaterium*	100	3.3 ± 0.4	1.4	—	0.6
	146786	KY646083	*α-Proteobacteria*	*Brevundimonas bullata*	99	3.1 ± 0.5	5.8	—	6.0
RO membrane									
	35813	KY393035	*β-Proteocbacteria*	*Hydrogenophaga pseudoflava*	100	22.9 ± 4.1	13.0	49.5	—
	88618	KR140227	*Actinobacteria*	*Subtercola frigoramans*	100	6.3 ± 1.1	55.2	8.1	—
	72044	NR125598	*α-Proteobacteria*	*Gemmobacter megaterium*	100	5.3 ± 0.6	2.2	1.6	—
	158580	NR109304	*α-Proteobacteria*	*Sphingobium fontiphilum*	100	4.7 ± 0.1	46.2	165.9	—
	144285	KX254991	*α-Proteobacteria*	*Sphingobium xenophagum*	100	3.8 ± 0.4	38.3	145.2	—

^a^All data were based on the average of 3 replicates, and the standard deviations are shown for relative abundances.

With respect to the OTUs of the membrane-attached biofilm microbiomes, it is noteworthy that the OTU 35813 identified as an autotrophic hydrogen-oxidizing bacterium *Hydrogenophaga pseudoflava* (KY393035, 100% similarity)^38^ exhibited quite high relative abundances (5.3–22.9% of the total) for both the MBR effluents of SWW and heavy oil-containing SWW, although it was quite minor (only 0.9–1.8% of the total) in the feed water microbiomes (see Supplementary Fig. S6). The relative abundances of this OTU in the biofilm microbiomes were 4.3–6.0 times and 13.0–49.5 times significantly higher than those in the feed water microbiomes for SWW and heavy oil-containing SWW, respectively. Although *Hydrogenophaga* spp. are not well known as the fouling-related bacteria, they have been frequently found from biofilm on RO membrane and RO feed water^33,36,39^. In addition, they have been detected from the stream biofilm formed under the turbulent flow condition^40^. From the physiological point of view, *Hydrogenophaga* spp. are able to synthesize polymeric substances^41^. It is most likely that the OTU 35813 grew autotrophically on RO membrane, thereby being directly responsible for the accumulation of extracellular polysaccharides and proteins in the biofilm (Figs 2 and 3). Apart from this autotrophic OTU, the OTU 138854 was phylogenetically assigned as *Blastochloris viridis* (LN907867, 96% similarity) (see Supplementary Table S4) that is able to produce proteins during the autotrophic growth under anaerobic conditions^42^, which corroborated the critical involvement of the autotrophic bacteria in the RO membrane fouling.

Furthermore, it is also noted that the common characteristics of the biofilm microbiomes for both the MBR effluents was the high dominance of oligotrophic bacteria. For SWW, the OTUs 163830 (*Azoarcus tolulyticus* [AF229876, 100% similarity]), 98583 (*Reyranella massiliensis* [NR116005, 100% similarity]), 171350 (*Hyphomicrobium vulgare* [NR104697, 99%]) and 137714 (*Gemmobacter lanyuensis* [KU991464, 100%]) have been characterized as freshwater bacteria^43–45^ (Table 2). For heavy oil-containing SWW, the OTUs 88618 (*Subtercola frigoramans* [KR140227, 100%]), 158580 (*Sphingobium fontiphilum* [NR109304, 100%]) and 179939 (*Aquimonas voraii* [KM199274, 100%]) were also the freshwater bacteria isolated from groundwater, freshwater spring and warm spring, respectively^46–48^ (Table 3). Most of these OTUs exhibited much low relative abundances in the feed water microbiomes, strongly suggesting the specific proliferation of these oligotrophic bacteria in the biofilm on RO membrane. In the RO system, the feed water was already purified by MBR and the nutrients for microbial growth have been limited considerably. Small amounts of the residual recalcitrant compounds in the MBR effluents and/or the nutrients synthesized by the co-existed autotrophic bacteria *Hydrogenophaga pseudoflava* (OTU 35813) and *Blastochloris viridis* (OTU 138854) may have served as the growth substrate for these oligotrophic bacteria of the membrane-attached biofilm microbiomes.

Identifying the key microorganisms that cause membrane fouling of RO system to treat MBR effluents is difficult, because the microbiomes on RO membrane are significantly influenced by the quality and volume of the feed water. The direct comparison of microbiomes of the feed water and membrane-attached biofilm indicated that microbial species affiliated within the genera *Hyphomicrobium* and *Sphingobium* were found specifically on the fouled RO membrane, emphasizing that these oligotrophic bacteria were involved in membrane fouling of RO system. Most importantly, the autotrophic bacteria *Hydrogenophaga pseudoflava* and *Blastochloris viridis*, the minor microbial species in the feed water, were newly identified as the abundant member on the fouled membrane. These autotrophic bacteria contributed to the formation of polysaccharide- and protein-rich biofilm on RO membrane, thereby causing membrane fouling of RO system.

## Electronic supplementary material

Supplementary Figures and Tables

## Data Availability

All the data generated and/or analyzed during this study are included in this article. The 16S rRNA gene sequence data were deposited in the sequence read archive in the DDBJ database (http://www.ddbj.nig.ac.jp/) under the accession number DRA006513.
