# Supplementary material for: Microbiomes and chemical components of feed water and membrane-attached biofilm in reverse osmosis system to treat membrane bioreactor effluents
Source: Sci Rep. 2018 Nov 14;8:16805. doi: 10.1038/s41598-018-35156-2 (PMC6235981; doi:10.1038/s41598-018-35156-2)
Supplement: Supplementary file 1 — Supplementary Figures and Tables [file 41598_2018_35156_MOESM1_ESM.pdf]

Supplementary materials for "Microbiomes and chemical components of feed water and membrane-attached biofilm in reverse osmosis system to treat membrane bioreactor effluents"

Tomohiro Inaba, Tomoyuki Hori*, Hidenobu Aizawa, Yuya Sato, Atsushi Ogata and Hiroshi Habe

Environmental Management Research Institute, National Institute of Advanced Industrial Science and Technology (AIST), 16-1 Onogawa, Tsukuba, Ibaraki 305-8569, Japan

*Corresponding author.
Tomoyuki Hori
Mailing address: Environmental Management Research Institute,
National Institute of Advanced Industrial Science and Technology (AIST), Onogawa 16-1, Tsukuba, Ibaraki 305-8569, Japan.
Phone: +81-29-849-1107
Fax: +81-29-861-8326
E-mail: hori-tomo@aist.go.jp

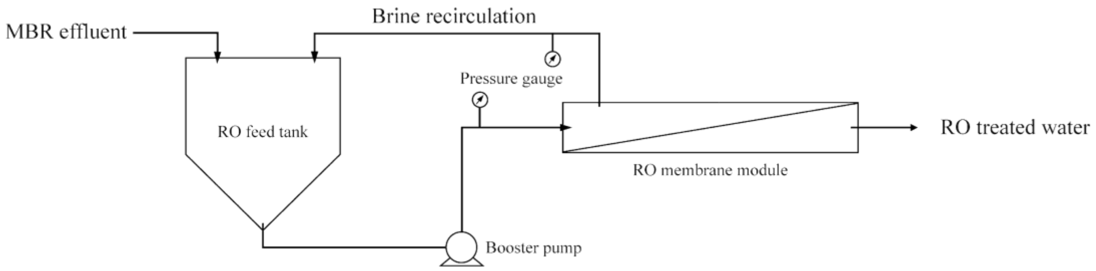

Figure S1. Schematic outline of RO system used in this study.

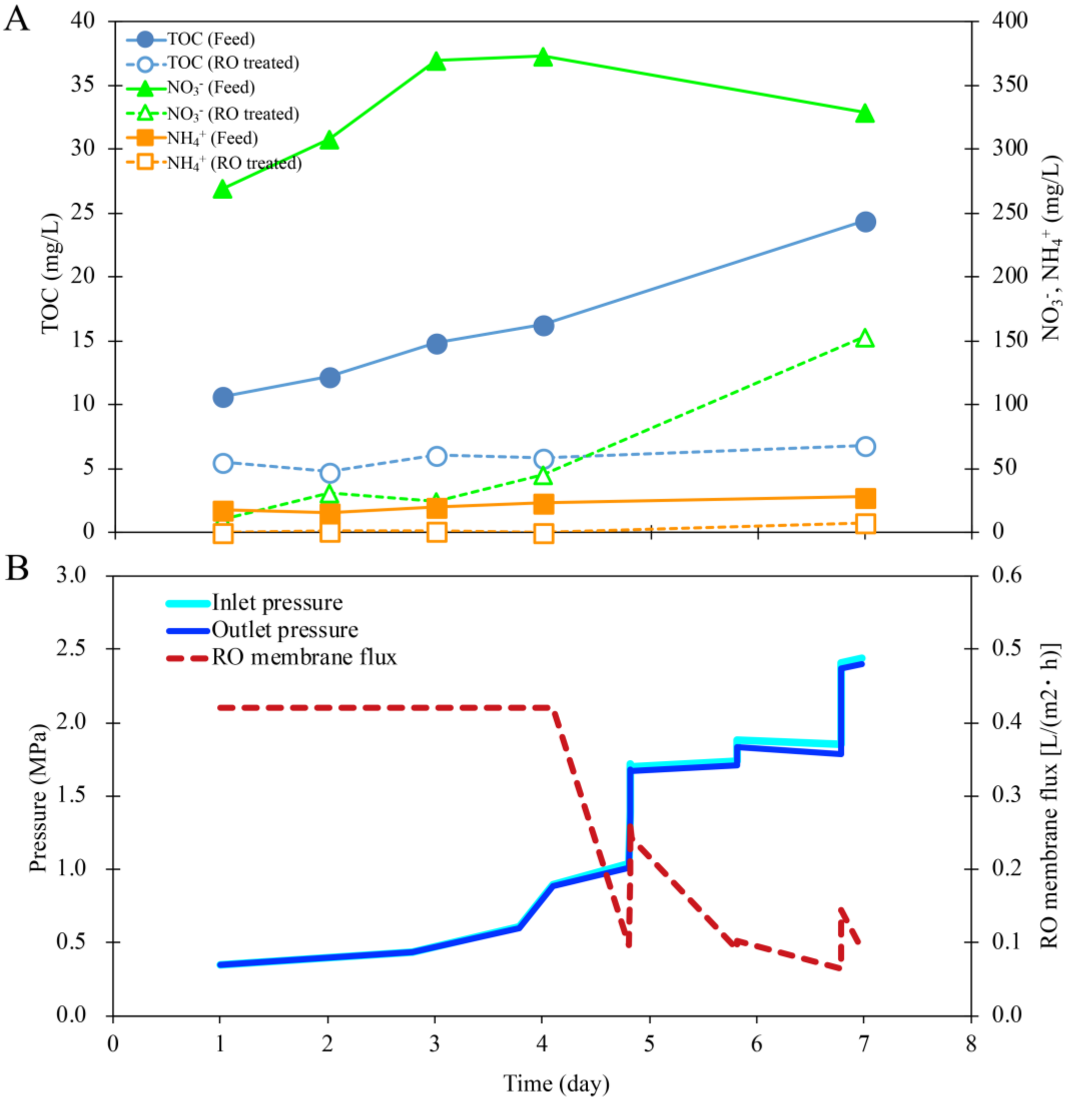

Figure S2. Physicochemical profiles during the operation of RO system for the MBR effluent of SWW. (A) Physicochemical profiles of feed water (closed symbols) and RO membrane-treated water (open symbols). (B) Transitions of operating pressures and RO membrane flux. Solid lines indicate the operating pressure and dashed line indicates the RO membrane flux.

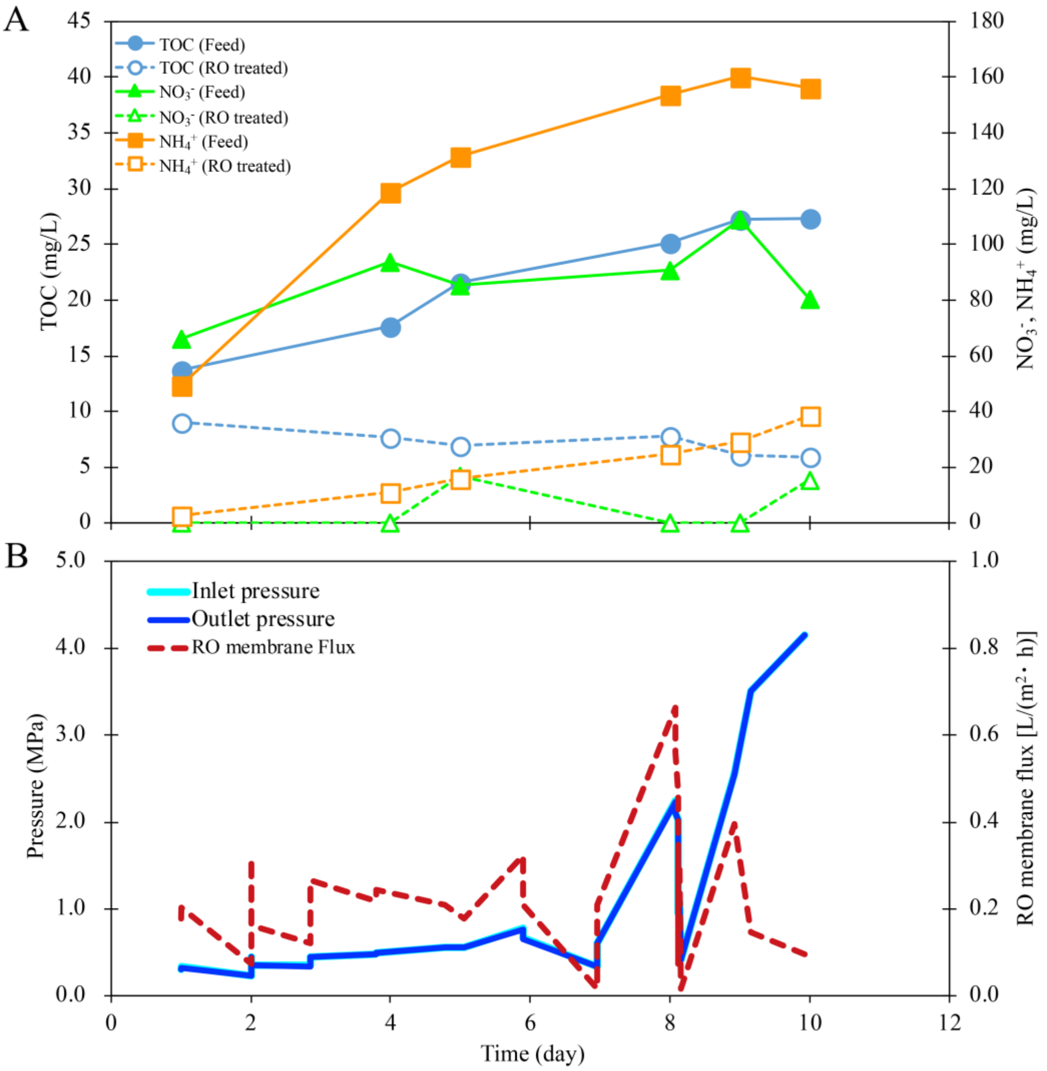

Figure S3. Physicochemical profiles during the operation of RO system for the MBR effluent of heavy-oil containing SWW. (A) Physicochemical profiles of feed water (closed symbols) and RO membrane-treated water (open symbols). (B) Transitions of operating pressures and RO membrane flux. Solid lines indicate the operating pressure and dashed line indicates the RO membrane flux.

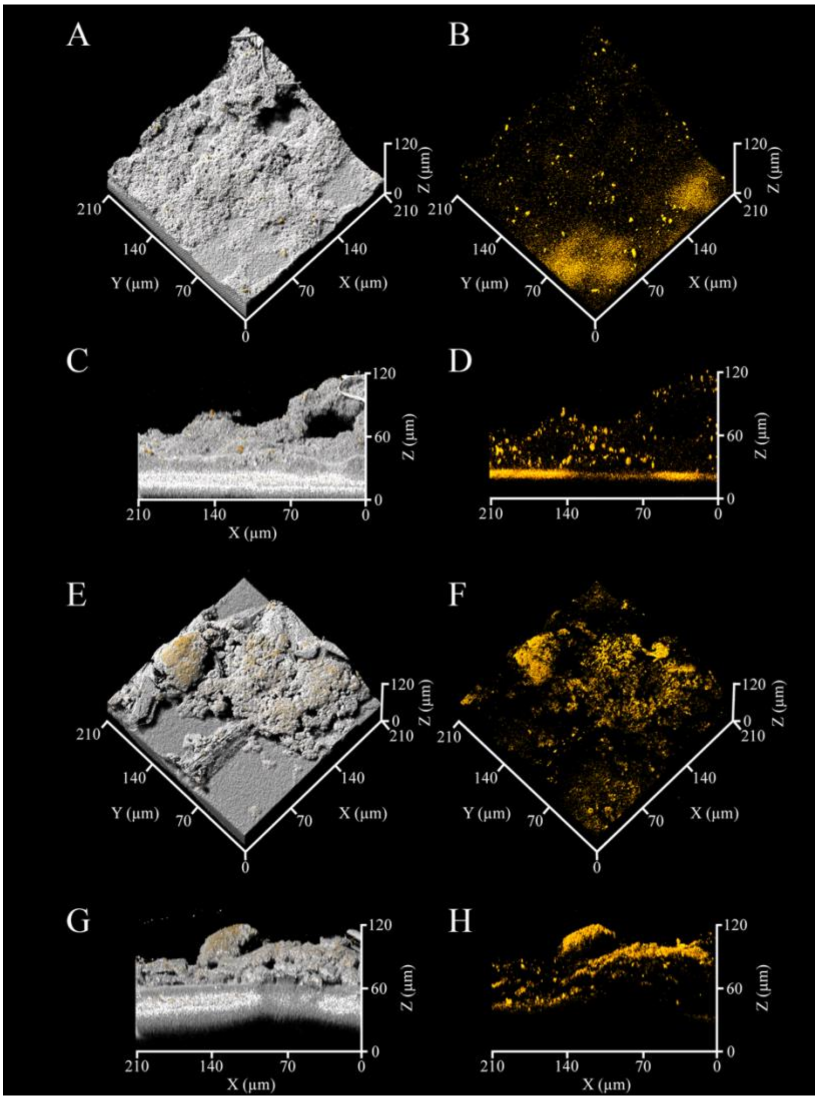

Figure S4. Proteins in the membrane-attached biofilms. Orange indicates proteins probed by SYPRO Ruby. Gray indicates the physical body reflected by light. The panels show the biofilms formed during RO membrane system to treat the MBR effluent of the SWW (A-D) and heavy oil-containing SWW (E-H). The fluorescent images of same region are also shown (B, D, F, H). At least 6 microscopic images at random points were taken per a sample and the representative images are shown.

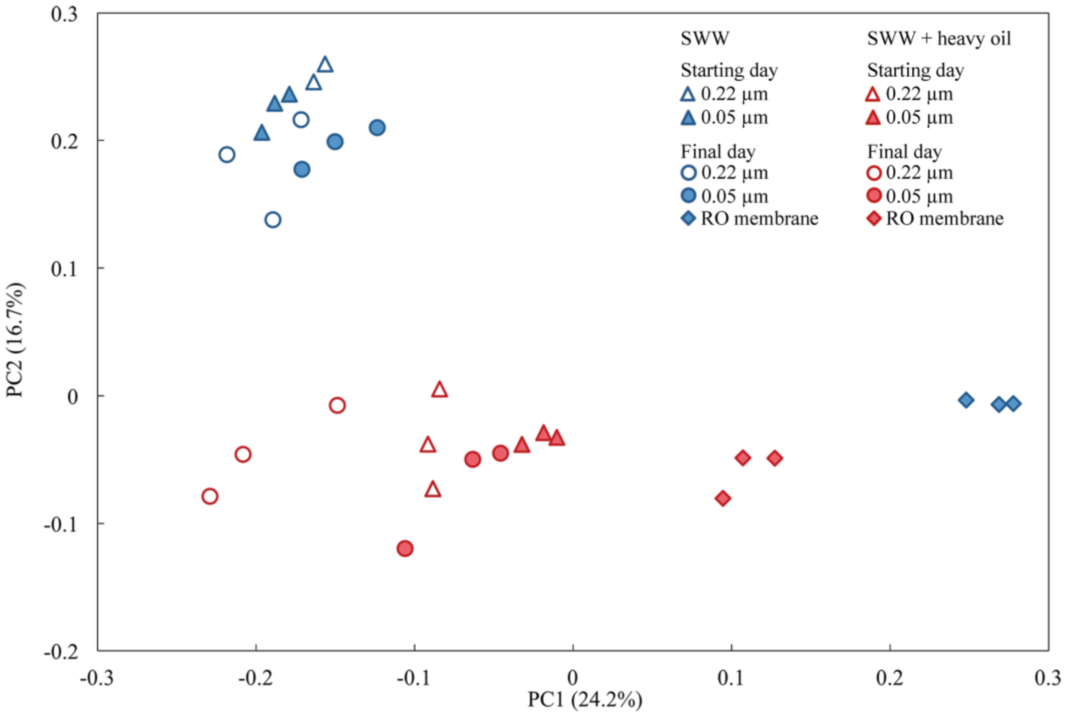

Figure S5. Comparison of the feed water and membrane-attached biofilm microbiomes. Principal coordinate analysis (PCoA) scatter plot of 16S rRNA genes obtained from Illumina sequencing is shown. The weighted UniFrac distances were calculated based on an equal number (n = 25,894) of sequences. Triangles and circles indicate the microbiomes of the feed water at the beginning and final days of the operation, respectively. The closed diamonds indicates membrane-attached biofilm microbiomes. The blue and red colors of symbols show those for the MBR effluent of the SWW and heavy oil-containing SWW, respectively.

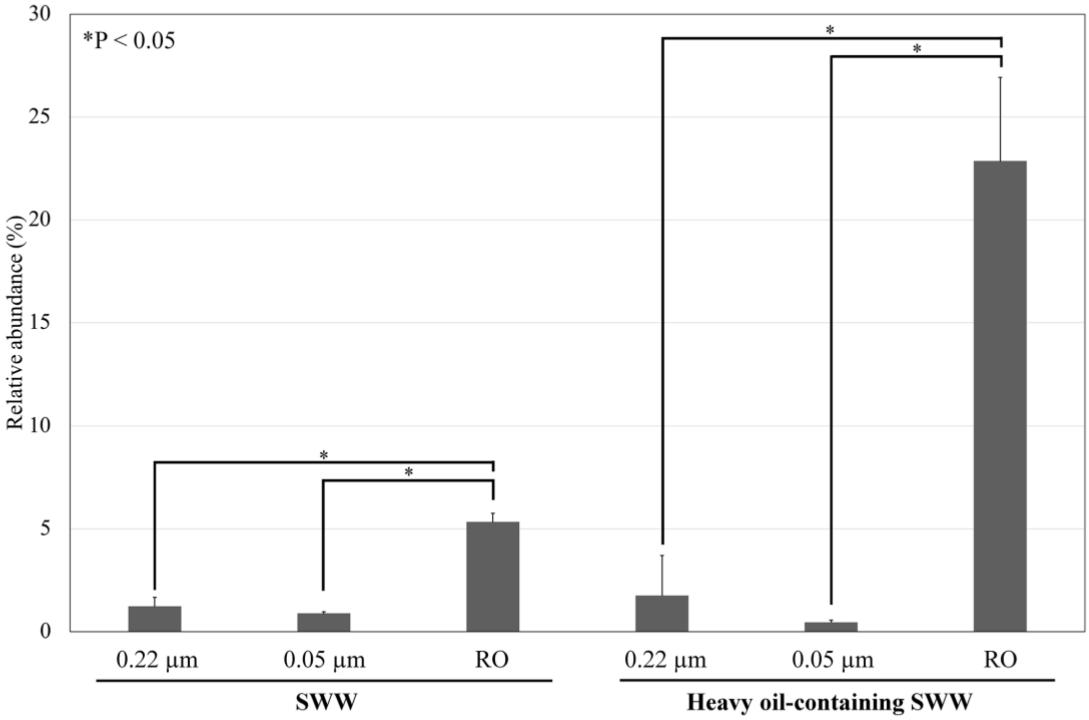

Figure S6. Relative abundances of the OTU 35813 (*Hydrogenophaga pseudoflava*) in microbiomes of the feed water and membrane-attached biofilms. These data were based on the average of 3 replicates and the standard deviations are indicated.

Table S1. Salt concentrations of RO feed water

Sample		NO_3^- (mg/L)	NH_4^+ (mg/L)	Cl^- (mg/L)	SO_4^{2-} (mg/L)	HPO_4^{2-} (mg/L)	Na^+ (mg/L)	Ca^{2+} (mg/L)	Mg^{2+} (mg/L)
SWW	Start	268.93	17.74	285.15	64.28	103.7	447.00	19.32	6.81
	End	329.12	28.01	549.66	108.76	196.61	988.00	28.94	11.60
Heavy oil-containing SWW	Start	66.07	49.30	271.60	33.61	93.97	488.1	8.9	6.3
	End	80.42	156.24	802.22	69.09	416.54	1688.60	26.40	6.50

Table S2. Summary of the Illumina sequencing data

Sample		Number of sequences	Number of OTUs	Cx[a]	Diversity indices[b]		
					Chao1	Shannon	1/Simpson
SWW	0.22-µm filter	32976±1452	284±44	0.997±0.0008	4027±865	3.83±0.55	5.55±1.46
	0.05-µm filter	38037±3472	319±23	0.997±0.0003	4432±1017	4.07±0.28	6.16±0.84
	RO membrane	100697±190 51	856±19	0.998±0.0003	11991±1448	7.44±0.17	45.43±4.64
Heavy oil-containing SWW	0.22-µm filter	44817±1768 7	373±133	0.997±0.0008	5301±1727	4.41±0.79	6.96±3.51
	0.05-µm filter	42978±2516	333±13	0.998±0.0001	4626±289	3.81±0.36	3.47±0.69
	RO membrane	91840±1241 7	815±97	0.998±0.0003	10559±707	6.83±0.24	19.33±4.46

[a]Calculated from the equation $C_X = 1 - (n/N)$, where "n" is the number of OTUs composed of singletons, and N is the total number of sequences.

[b]Each index was calculated based on an equal number of sequences (n = 12,556). All data were based on the average of 3 replicates and the standard deviations are shown.

Table S3. Sequence data summary of feed water at the beginning of RO system operation

Sample		Number of sequences	Number of OTUs	Cx[a]	Diversity indices[b]		
					Chao1	Shannon	1/Simpson
SWW	0.22-µm filter	54972±11796	370±1	0.998±0.0010	4561±580	4.18±0.28	5.16±0.71
	0.05-µm filter	37525±3178	322±31	0.997±0.0001	4230±542	4.55±0.19	7.44±0.82
Heavy oil-containing SWW	0.22-µm filter	28556±3413	356±10	0.996±0.0009	5621±809	5.53±0.32	11.50±2.17
	0.05-µm filter	52144±28401	555±127	0.997±0.0010	8516±1245	6.39±0.24	24.57±3.30

[a]Calculated from the equation $C_X = 1 - (n/N)$, where "n" is the number of OTUs composed of singletons, and N is the total number of sequences.

[b]Each index was calculated based on an equal number of sequences (n = 12,556). All data were based on the average of 3 replicates and the standard deviations of are shown.

Table S4. The top 6 to 10 abundant OTUs in microbiomes of the feed water and membrane-attached biofilm for SWW

OTU ID	Accession Number	Bacteria Class	Bacteria Species	Identity (%)	Relative abundance (%)[a]	Fold change relative to 0.22 µm[a]	Fold change relative to 0.05 µm[a]	Fold change relative to RO[a]
0.22 µm								
98583	NR116005	*α-Proteobacteria*	*Reyranella massiliensis*	100	3.3±0.5	−	1.3	0.4
35813	KY393035	*β-Proteocbacteria*	*Hydrogenophaga pseudoflava*	100	1.2±0.4	−	1.4	0.2
83690	KY576007	*α-Proteobacteria*	*Brevundimonas denitrificans*	100	1.2±0.3	−	0.3	0.4
27876	NR125542	*β-Proteocbacteria*	*Limnohabitans parvus*	100	0.9±0.3	−	1.9	1.8
119287	NR118485	*α-Proteobacteria*	*Caulobacter daechungensis*	100	0.6±0.1	−	0.4	2.7
0.05 µm								
83690	KY576007	*α-Proteobacteria*	*Brevundimonas denitrificans*	100	4.0±0.3	3.4	−	1.3
98583	NR116005	*α-Proteobacteria*	*Reyranella massiliensis*	100	2.5±0.2	0.8	−	0.3
74997	KY241478	*β-Proteocbacteria*	*Limnobacter thiooxidans*	100	2.3±0.1	0.5	−	26.5
119287	NR118485	*α-Proteobacteria*	*Caulobacter daechungensis*	100	1.7±0.1	2.6	−	7.1
35813	KY393035	*β-Proteocbacteria*	*Hydrogenophaga pseudoflava*	100	0.9±0.1	0.7	−	0.2
RO membrane								
78900	NR074262	*α-Proteobacteria*	*Parvibaculum lavamentivorans*	100	3.2±0.1	24.5	28.7	−
83690	KY576007	*α-Proteobacteria*	*Brevundimonas denitrificans*	100	3.1±0.1	2.7	0.8	−
138854	LN907867	*α-Proteobacteria*	*Blastochloris viridis*	96	3.1±0.4	211.8	184.3	−
20440	NR147759	*α-Proteobacteria*	*Devosia humi*	100	3.0±0.3	20.9	32.5	−
106754	KC534373	*α-Proteobacteria*	*Hyphomonas johnsonii*	99	2.8±0.3	52.3	62.3	−

[a]All data were based on the average of 3 replicates and the standard deviations are shown for relative abundances.

Table S5. The top 6 to 10 abundant OTUs in microbiomes of the feed water and membrane-attached biofilms for the heavy oil-containing SWW

OTU ID	Accession Number	Bacteria		Identity (%)	Relative abundance (%)[a]	Fold change relative to		
		Class	Species			0.22 μm[a]	0.05 μm[a]	RO[a]
0.22 μm								
4145	NR024752	*Bacilli*	*Paenibacillus koleovorans*	99	2.6±0.6	−	208.0	4.5
72044	NR125598	*α-Proteobacteria*	*Gemmobacter megaterium*	99	2.4±0.5	−	0.7	0.4
12685	KX980435	*γ-Proteobacteria*	*Pseudomonas anguilliseptica*	100	2.0±1.8	−	13.5	5.2
35813	KY393035	*β-Proteocbacteria*	*Hydrogenophaga pseudoflava*	100	1.8±1.9	−	3.8	0.08
115593	NR109520	*Flavobacteriia*	*Flavobacterium marinum*	98	1.7±0.5	−	12.0	31.4
0.05 μm								
76428	KF528160	*Sphingobacteriia*	*Solitalea canadensis*	89	2.6±0.8	8.5	−	35.9
119287	NR118485	*α-Proteobacteria*	*Caulobacter daechungensis*	100	2.2±0.3	8.6	−	2.2
133530	KY643712	*γ-Proteobacteria*	*Pseudomonas stutzeri*	100	1.9±0.1	0.1	−	78.4
102392	KY021738	*α-Proteobacteria*	*Paracoccus alkenifer*	97	1.6±0.4	1.0	−	35.1
148841	KC634318	*γ-Proteobacteria*	*Pseudomonas caeni*	99	1.5±0.1	79.0	−	NA
RO membrane								
83690	KY576007	*α-Proteobacteria*	*Brevundimonas denitrificans*	100	3.4±0.2	1.1	0.5	−
179939	KM199274	*γ-Proteobacteria*	*Aquimonas voraii*	99	3.2±0.3	8.9	5.7	−
101077	NR133714	*Bacteroidia*	*Carboxylicivirga mesophila*	87	2.8±0.2	NA	588.9	−
147154	AB219940	*α-Proteobacteria*	*Sphingosinicella microcystinivorans*	100	2.7±0.4	91.3	190.2	−
98520	NR145906	*Actinobacteria*	*Leucobacter zea*	99	2.4±0.2	19.2	8.1	−

NA, Not applicable.

[a]All data were based on the average of 3 replicates and the standard deviations are shown for relative abundances.
